# Investigation of the Mechanical Properties of Mn-Alloyed Tin-Silver-Copper Solder Solidified with Different Cooling Rates

**DOI:** 10.3390/ma13225251

**Published:** 2020-11-20

**Authors:** Tamás Hurtony, Oliver Krammer, Balázs Illés, Gábor Harsányi, David Bušek, Karel Dušek

**Affiliations:** 1 Department of Electronics Technology, Faculty of Electrical Engineering and Informatics, Budapest University of Technology and Economics, 1111 Budapest, Hungary; hurtony@ett.bme.hu (T.H.); billes@ett.bme.hu (B.I.); harsanyi@ett.bme.hu (G.H.); 2 Department of Electrotechnology, Faculty of Electrical Engineering, Czech Technical University in Prague, 166 36 Prague 6, Czech Republic; busekd1@fel.cvut.cz (D.B.); dusekk1@fel.cvut.cz (K.D.)

**Keywords:** lead-free soldering, manganese alloying, mechanical testing, hardness testing, structural analysis

## Abstract

Manganese can be an optimal alloying addition in lead-free SAC (SnAgCu) solder alloys because of its low price and harmless nature. In this research, the mechanical properties of the novel SAC0307 (Sn/Ag0.3/Cu0.7) alloyed with 0.7 wt.% Mn (designated as SAC0307-Mn07) and those of the traditionally used SAC305 (Sn96.5/Ag3/Cu0.5) solder alloys were investigated by analyzing the shear force and Vickers hardness of reflowed solder balls. During the preparation of the reflowed solder balls, different cooling rates were used in the range from 2.7 K/s to 14.7 K/s. After measuring the shear force and the Vickers hardness, the structures of the fracture surfaces and the intermetallic layer were investigated by SEM (Scanning Electron Microscopy). The mechanical property measurements showed lower shear force for the SAC0307-Mn07 alloy (20–25 N) compared with the SAC305 alloy (27–35 N), independent of the cooling rate. However, the SAC0307-Mn07 alloy was softer; its Vickers hardness was between 12 and 13 HV, whereas the Vickers hardness of the SAC305 alloy was between 19 and 20 HV. In addition, structural analyses revealed rougher intermetallic compound layers in the case of the SAC0307-Mn07 alloy, which can inhibit the propagation of cracks at the solder–substrate interface. These two properties of SAC0307-Mn07 alloy, the softer nature and the rougher intermetallic layer, might result in better thermomechanical behavior of the solder joints during the lifetime of electronic devices.

## 1. Introduction

The most frequently used soldering technology for the automated assembly of electronic devices is the reflow soldering technique [1], which typically utilizes forced convection or vapor phase for the transferring of heat to the assembly [2,3]. The compulsory introduction of lead-free alloys in assembly technologies in the EU and the restriction of hazardous substances (RoHS Directive 2002/95/EC, RoHS 2011/65/EU) forced the electronic industry to eliminate lead-bearing solder alloys in many applications, which resulted in the intensive development of new solder alloys.

The changeover was not straightforward; the reflow profile became more difficult to set appropriately because of the smaller difference in temperature between the onset of alloy melting and the heat resistance of components [4]. Additionally, the lead-free alloys that were initially developed exhibited lower performance than the conventional lead-bearing solders from both mechanical and reliability points of view [5]. Recently, the widespread lead-free alloy utilized in the industry is the SAC305. The major weakness of this alloy is its lower strength in dynamic testing, such as in drop-shock tests. The risk of formation of “shrinkage” defects after soldering is also larger, when the cooling rate is lower than ~1.5 K/s at solder joints of especially BGA (ball grid array) or TH (through-hole) components. The reliability problems are caused by the relatively high silver content, which is regularly hyper-eutectic (>1.35 wt.%) in these alloys [6]. Even if the reflow profile is precisely monitored [7,8] during the soldering process, and the volume of the solder paste is thoroughly adjusted [9], electronic assemblies and devices can exist in which reliability criteria cannot be met. For instance, in a harsh operating environment, such as overpressure [10,11], the joints prepared from SAC305 alloy can underperform. Consequently, research on new lead-free solder alloys is still ongoing in microelectronics. Lower silver content alloys, such as Sn99/Ag0.3/Cu0.7 (SAC0307), form fewer primary Ag_3_Sn intermetallic plates when undercooled (this lowers the risk of “shrinkage” defects), but the mechanical properties also lag behind the SAC305 alloy. That is the reason for ongoing research regarding the alloying of SAC0307.

Many researchers have investigated the effect of adding bismuth or nickel to the plain SAC alloys to improve their wettability [12] and (thermo-)mechanical properties. Chen et al. researched the tensile behavior of SAC solders alloyed with bismuth [13]. They demonstrated that bismuth incorporates into the Sn as a solid-solution, and its addition as the second phase can slow down the mobility of dislocations effectively so that the tensile strength of SAC alloys increases observably. The addition of bismuth into the SAC alloy can also decrease the alloy melting point extensively, to as low as 138 °C [14], which allows soldering onto temperature-sensitive substrates. Liu et al. analyzed the hardness and shear strength of tin-bismuth alloys, in which SnAgCu particles were added in various weight percentages [15]. They showed that the addition of SnAgCu particles over 8% wt.% aids the precipitation of submicron-sized bismuth grains in the tin phase. This resulted in an increase in hardness values but lowered the strength of solder joints. Hardness and microhardness variations against various effects, such as contact potential difference or electric potential [16], and surface treatments, such as electron-plasma treatment [17], have also been investigated for different alloys.

Other metals that are typically used in SAC alloys are nickel, antimony, and zinc to lower the melting temperature [18] or to enhance the mechanical, structural properties of the alloys. Roa et al. investigated SAC alloys containing Zn [19]. They showed that Zn refined the grain structure of the solder joints and yielded less dendritic precipitation of Ag_3_Sn intermetallic compounds. Benabou et al. analyzed the effect of adding nickel or antimony [20]. They revealed that these alloys under test suppress the growth of both Cu_6_Sn_5_ and Cu_3_Sn intermetallic layers during high-temperature storage life tests.

Recently, materials such as manganese, boron, cerium and titanium have been added to the plain SAC alloys to improve their mechanical response for dynamic loads, such as drop-shocks or thermal shocks. Wang et al. revealed that adding boron can reduce the rate of growth of interfacial intermetallic layers, which can slow down crack propagation over the lifetime of electronics [21]. Because of the reasonable price and harmless nature, manganese can be an optimal alloying element for the SAC solder alloys. Mn reportedly refines the grain structure in the SAC solder alloys when added either in the nanoparticle [22] or in the alloyed form [23]. Both at soldering temperature and below that, the solubility of Mn in the Sn solder alloy is low. Consequently, Mn-containing micro precipitation can form inside the solder joint, even by relatively low concentration. The bismuth-containing precipitations can act as crystallization centers during the solidification, thus refining the structure of solder joints. Alloys with finer microstructure can bear intensive and dynamic external mechanical loads, such as drop tests, better [24]. Liu et al. analyzed the reliability of lead-free alloys, in which manganese and cerium were added [24]. They proved that these metals can increase the drop shock reliability of lead-free solder joints. Song et al. also confirmed by ball impact testing the improved mechanical behavior of alloys with manganese dopants [25]. Lin et al. measured the elongation and Young’s modulus of SAC solders alloyed with manganese and titanium [23] and showed an increase in the elongation and a decrease in the Young’s modulus. Manganese was also utilized in nanoparticle form [22]. Tang et al. found that the optimal weight percentage of manganese nanoparticles is 0.05 wt.% to enhance the wettability of SAC alloys. The addition of minor composing elements allows the amount of silver in SAC solders to be reduced, which can result both in reliability increase [26] and significant cost reduction.

The mechanical and thermomechanical behaviors of SAC solders alloyed with various materials have been comprehensively investigated, and manganese addition is a sound research direction. However, there are areas that have not yet been addressed, e.g., the effect of cooling rates on the mechanical and hardness properties of Mn-alloyed SAC solders. Investigating these properties is the key for the optimization and proper adjustment of reflow profiles for these types of alloys.

## 2. Materials and Methods

**Figure 1:**
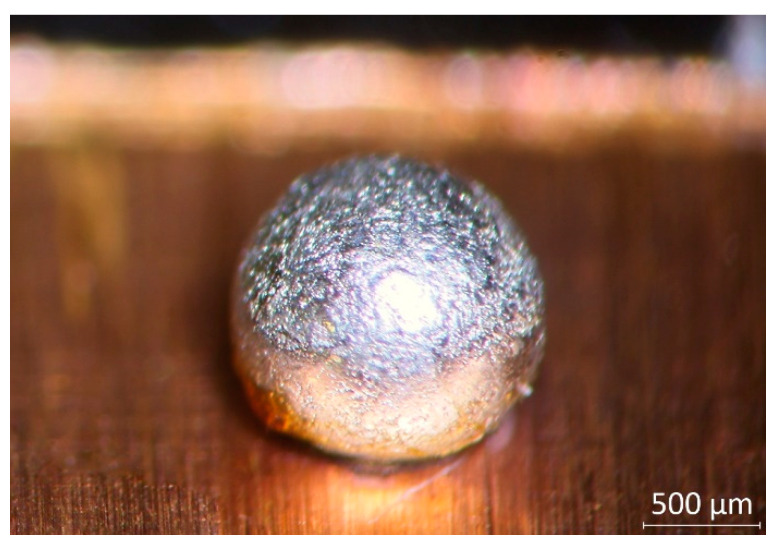
A reflowed solder ball on the copper substrate.

Two solder alloys were chosen for the experiments: SAC305 (Sn96.5/Ag3/Cu0.5), which is traditionally used in the industry, and a low-silver-content Sn/Ag0.3/Cu0.7 (SAC0307) alloyed with 0.7 wt.% Mn (referred to as SAC0307-Mn07). The aim was to investigate whether Mn alloying improves the mechanical and/or structural properties of solder joints. In this experiment, solder balls with the investigated alloys were prepared onto copper substrates. The SAC0307-Mn07 solder alloy was obtained from Metalloglobus Zrt. in wire form with a diameter of 1 mm. Equidistant laser marks were engraved onto the solder wire (along which the wire was cut) to prepare solder preforms with equal volume. The solder-preforms were cleaned in a sonic bath and stored under isopropanol before being used. The substrates were prepared from 0.2 mm thick copper sheets, which had been cut into 10 × 5 mm^2^ pieces. After cleaning, the total surface of the substrate was covered with Kapton^®^ tape. Circle-shaped pads were then formed by cutting the Kapton^®^ tape with a UV-Nd:YAG laser. The diameter of the solder pads was chosen so that the volume-to- surface ratio of the solder bumps would not limit the diffusion of the substrate Cu atoms into the solder bulk. No diffusion barrier underlayer was deposited between the solder and the substrate, thus modelling printed wiring substrates with copper pads and thin coatings, like OSP (Organic Solderability Preservative), ImSn (immersion tin), or ImAg (immersion silver). The fixed volume and the solder mask defined solder pads resulted in spherically symmetric solder balls approximately 2 mm in diameter (Figure 1). The shape and size of the solder ball were selected to be compatible with the testing methods used (hardness and shear force measurement).

The cylindrical symmetricity of the solder ensured uniform heat distribution all over the solder interface. Similar morphology of the intermetallic layer was expected in the lateral direction.

**Figure 2:**
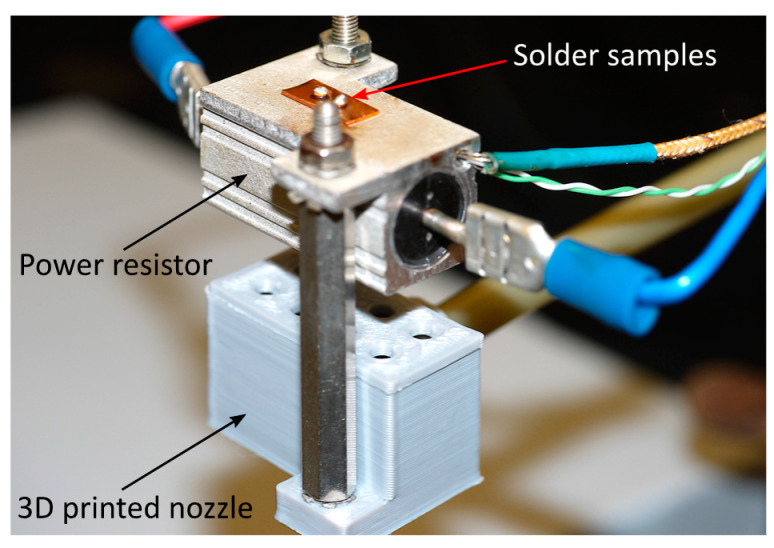
The custom-made hot plate for preparing the solder ball samples with different cooling rates.

**Figure 3:**
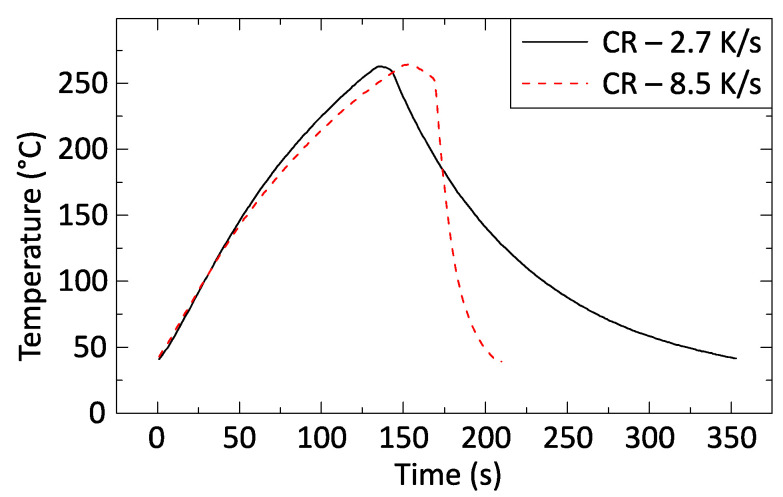
Reflow profiles with different cooling rates.

The solder samples were reflowed on a hot plate specifically designed to have minimal thermal inertia. The heat source was a power resistor with finned aluminum housing. Because a thermal interface material was used between the sample and the hotplate, the temperature difference between them is considered to be minimal. A hole was drilled into the housing up to the center of the component. K-type thermocouples were placed into this hole to monitor the temperature of the samples. The varied cooling rate was achieved by forced convection. A 3D-printed nozzle was attached to the bottom of the hotplate, and the airstream was constrained to the required pressure through a regulator (Figure 2). Table 1 summarizes the cooling rates used and the corresponding regulator pressure values, and Figure 3 illustrates two typical profiles with cooling rates of 2.7 and 8.5 K/s.

The solder preforms were placed onto the hotplate when its temperature reached 230 °C. After 3–5 s, the solder melted and wetted the solder pads. When the temperature of the hot plate reached 260 °C, the power supply was turned off, and the pressurized airstream was released.

The solidified solder balls were then sheared with Dage 4000 Multi-Purpose Shear equipment (Nordson Dage, Aylesbury, UK) with a 50 N load cell and 100 µm/s travel speed. The sheared balls were embedded into two-component epoxy resin and cross-sectioned. The hardness of the cross-sectioned balls was measured with a Struers Duramin II tester (Detroit, MI, USA). An HV0.5 load (4.903 N) and 10 s of dwell time were used. The cross-sectional and fracture surface of the sheared components were inspected with a FEI Inspect S50 Scanning Electron Microscope (SEM) (Hillsboro, OR, USA) and Bruker Quanta 200 Energy-dispersive X-ray spectroscopy (EDS) (Billerica, MA, USA). Additionally, the remaining solder alloy on the fracture surface after shearing was removed with a selective electrochemical etching process, described in [27]. With a standard three-electrode electrochemical cell geometry, the pure tin phase was selectively extracted from the solder joints, keeping the copper and the intermetallic phases intact. When the current reached a threshold value (100 µA in our case) in chronoamperometry mode, the etching process was terminated because all the removable pure tin phase had already been removed, exposing the intermetallic layer on the solder–substrate interface.

## 3. Results and Discussion

**Figure 4:**
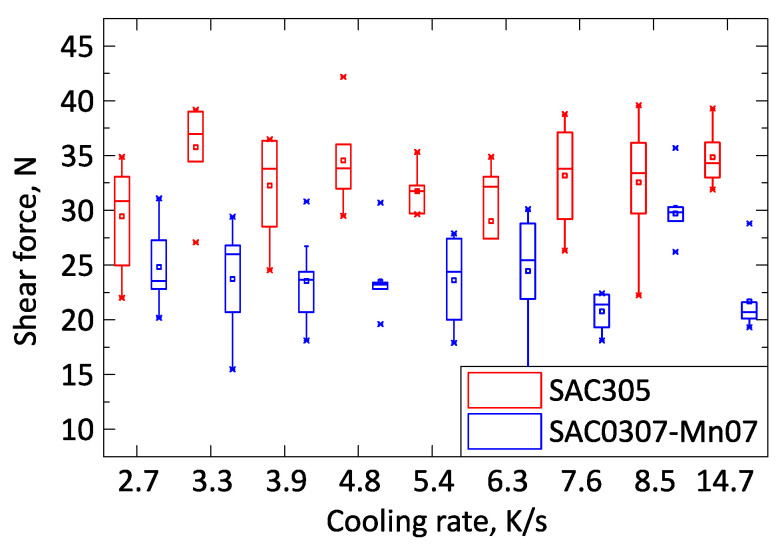
Shear force of SAC305 and SAC0307-Mn07 samples.

First, the mechanical properties of the samples were analyzed. No significant difference in the shear force was observed between the solder samples solidified with different cooling rates (Figure 4). The shear force of the SAC305 solder joints was systematically higher, approximately by 30%, than that of the SAC0307-Mn07 solder joints.

**Figure 5:**
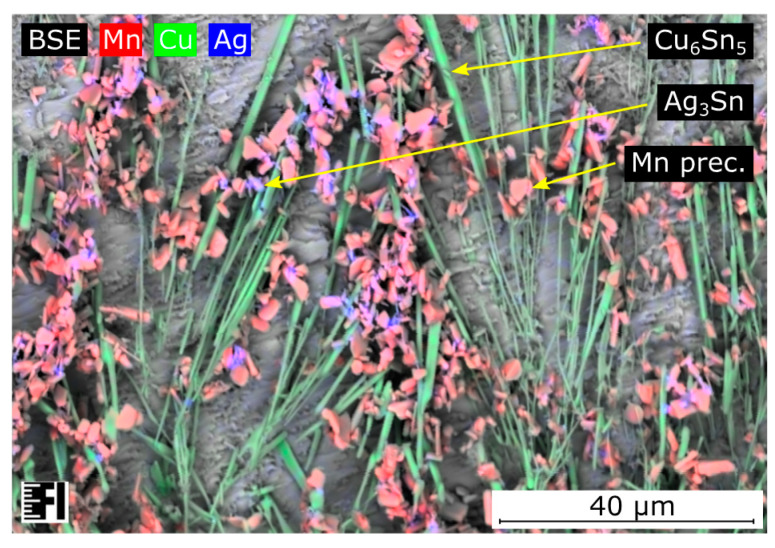
SEM-EDS material composition map of SAC0307-Mn07 solder joint. Mn precipitates are dispersed inside the bulk, usually around the Ag_3_Sn intermetallic compounds.

During the shear test, all samples were separated from the substrate inside the solder bulk close to the solder–substrate interface. The strength of the bulk material appeared to be the weakest. The Mn precipitates caused grain refinement of β-tin grains inside the solder bulk as reported in the literature [20,21]. However, the finer grain structure did not necessarily yield a higher shear force. Although the number of grain boundaries increased, there was no reinforcement material between the dendritic arms of the β-tin phase. The Mn precipitates were usually granular submicron particles (Figure 5), which do not have significant mechanical supporting contributions inside the solder bulk. The Ag_3_Sn intermetallic compounds and Mn particles were always in the same region, while the Ag_3_Sn likely nucleated on the surface of Mn initiators. The Mn particles might indirectly have had some influence on the overall strength of the alloy while they facilitated the nucleation of the Ag_3_Sn intermetallic compounds. Inside the higher silver-content SAC305 solder joints, the Ag_3_Sn intermetallics were distributed homogenously inside the solder bulk, supporting the joint better than in the case of SAC0307-Mn07 solder joints. Nevertheless, the relatively high silver content appeared unfavorable from the point of view that primary Ag_3_Sn intermetallic plates can form at lower cooling rates. Since these intermetallic compounds are reported to be brittle, the Ag_3_Sn plates promote the propagation of the cracks inside the solder joint.

**Figure 6:**
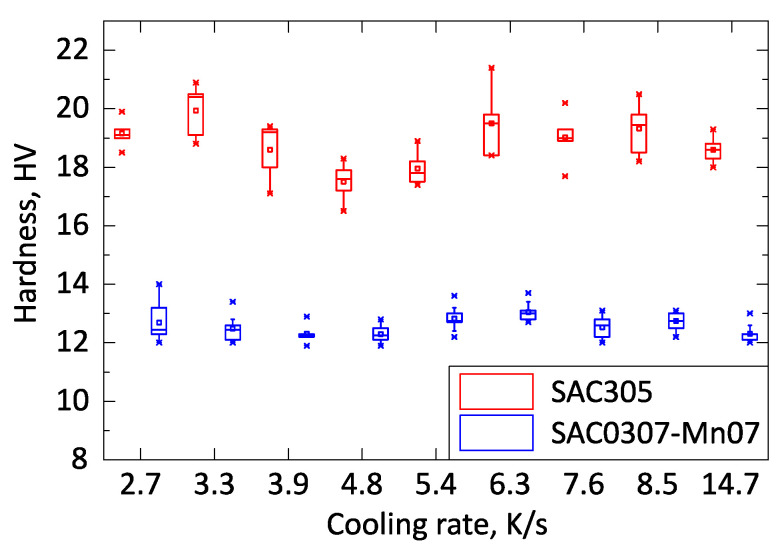
Hardness values of SAC305 and SAC0307-Mn07 samples.

The hardness test showed remarkable low standard deviation, and the results were in good correlation with the shear force measurement. No significant hardness change was observed in the investigated cooling rate range of 2.7–14.7 K/s (Figure 6). The SAC305 samples had higher hardness values than the SAC0307-Mn07 samples by a factor of approximately 40%. The SAC0307-Mn07 solder alloy is significantly softer than SAC305. More rigid joints are less immune to cyclic external load [28], like vibration or cyclic thermal load, cyclic thermal shock, whereas softer alloys (like the SAC0307-Mn07) can bear better these loads. All in all, the SAC0307-Mn07 samples showed lower shear force in as-reflowed state (testing right after the soldering), but their thermomechanical properties might be better because of the lack of Ag_3_Sn intermetallic plates formation and their softer nature.

**Figure 7:**
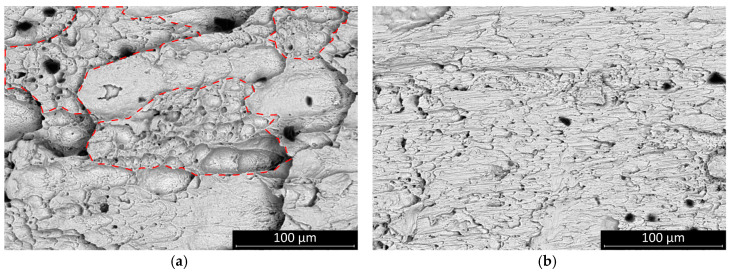
Fracture surfaces of the samples cooled at the rate of 2.7 K/s: (**a**) SAC305, areas of plastic deformation are indicated by dashed lines as examples; (**b**) SAC0307-Mn07, significant plastic deformation areas were not observed.

The fracture surface of the sheared joints was inspected by scanning electron microscopy. All fractures occurred inside the solder bulk close to the solder–substrate interface. The fractures of the SAC305 solder joints appear more ductile. The surface features suggest that the solder had been loaded up to the threshold of the plastic deformation of the solder bulk. The SAC0307-Mn07 was also rather ductile, but the total amount of plastic deformation area was smaller than it was at the SAC305 (Figure 7—areas are indicated by dash lines).

**Figure 8:**
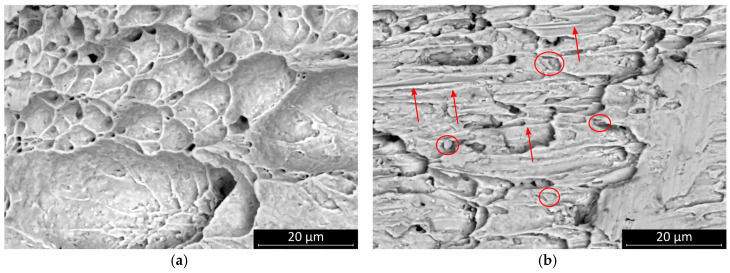
SEM micrographs of the fracture surface of samples cooled at 2.7 K/s: (**a**) SAC305; (**b**) SAC0307-Mn07, the Mn precipitates are marked by red circles, and the scratch lines are indicated with red arrows.

**Figure 9:**
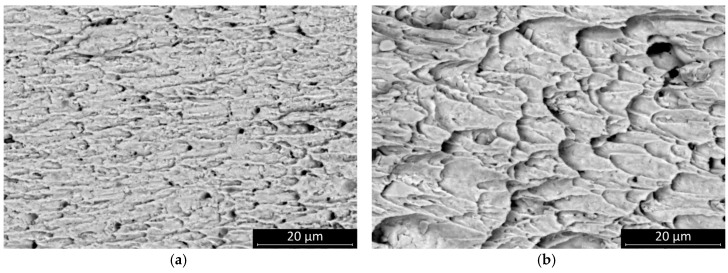
SEM micrographs of the fracture surface of samples cooled at the rate of 14.7 K/s: (**a**) SAC305; (**b**) SAC0307-Mn07.

This result suggests that the SAC0307-Mn07 solder joints started to creep at a relatively lower load. Mn-containing precipitates were observed on the fracture surface. These granular objects were most smeared during the shear motion at the fracture plane, and they created parallel scratch marks on the surface. The presence of these phases probably created a slip plane along which the solder joint was separated at a relatively smaller shear force value (Figure 8). No significant change was observed in the characteristic features of the fracture surface of SAC0307-Mn07 solder joints at different cooling rates, but the scratch lines caused by Mn precipitates disappeared. At a higher cooling rate, the SAC305 solder joints showed less ductile behavior, and the portion of the plastic deformation region became less dominant (Figure 9). The morphology of the fracture surface significantly changed in the case of SAC305 solder joints at higher cooling rates. On the other hand, no characteristic deviation was observed in SAC0307-Mn07 samples.

For further analyses, the remaining solder alloy on the fracture surface after shearing was removed with a selective electrochemical etching process, described in [27]. The etching exposed the intermetallic layer on the solder–substrate interface.

**Figure 10:**
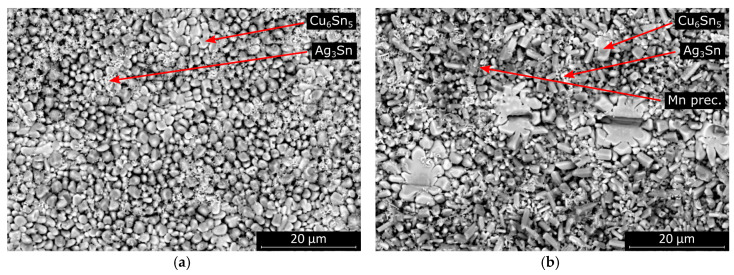
SEM micrographs of the exposed intermetallic layer of the samples cooled at the rate of 2.7 K/s: (**a**) SAC305; (**b**) SAC0307-Mn07.

**Figure 11:**
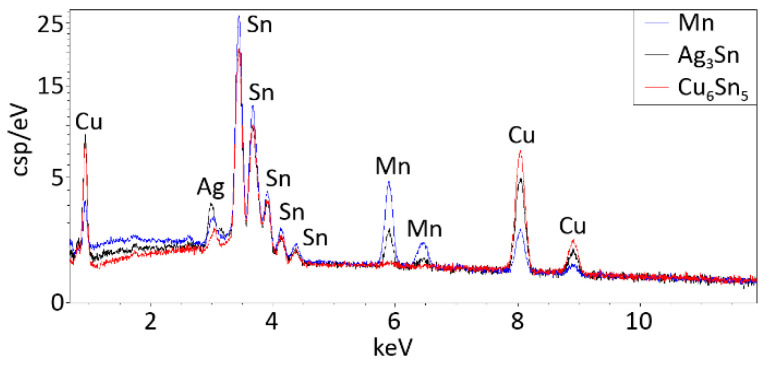
EDS spectra for the intermetallic compounds and for the Mn precipitate, which are indicated by arrows in Figure 10b.

The morphology of the intermetallic layer in the SAC0307-Mn07 solder joints significantly differed from that in the SAC305. Almost the entire interface was covered with highly elongated intermetallic grains. The more rounded scallop-type intermetallic grains are energetically favorable in the case of wetting reaction. Therefore, secondary crystallization of the intermetallic grains onto the surface of the originally rounded grains is suspected in the SAC0307-Mn07 solder joints (Figure 10). The EDS spectra for the intermetallic compounds and for the Mn precipitate (pointed by arrows in Figure 10b) are illustrated in Figure 11.

**Figure 12:**
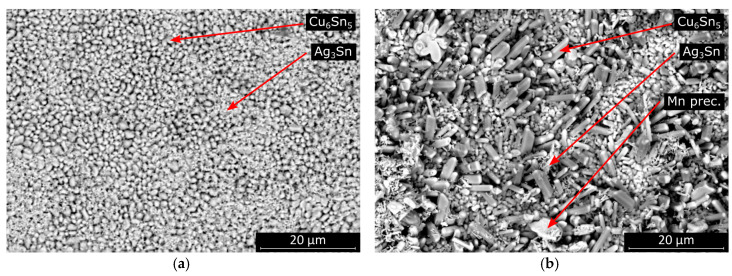
SEM micrographs of the exposed intermetallic layer of the samples cooled at the rate of 14.7 K/s: (**a**) SAC305; (**b**) SAC0307-Mn07.

The morphology was similar, but the average intermetallic grain size was significantly smaller at higher cooling rates in the case of SAC305 solder joints (Figure 12). The morphology of the SAC0307-Mn07 solder joints also changed slightly at higher cooling rates. The fraction of the area covered by more elongated intermetallic grains became smaller at higher cooling rates. Between the oblong grains, scallop-type grains covered the entire solder–substrate interface.

The rougher intermetallic layer at the interface is favorable since the propagation of cracks is inhibited by the rougher interface. Typically, the gaps between the intermetallic layer grains are filled during an aging process (like high-temperature storage life test). The rougher surface of intermetallic layers may yield less susceptibility to failure mode caused by the aging of the samples.

## 4. Conclusions

The mechanical properties of SAC305 and Mn-alloyed Sn/Ag0.3/Cu0.7 solders were investigated by using different cooling rates (2.7–14.7 K/s). It was found that the mechanical properties were not significantly changed over the different cooling rates, but differences in the microstructures were observed between the alloys. The SAC0307-Mn07 alloy behaved systematically softer against mechanical load than the SAC305 alloy, which makes the SAC0307-Mn07 a promising creep-resistant alloy for long-term operation. In addition, the morphology of the intermetallic layer at the solder–substrate interface was also significantly different in the case of the SAC0307-Mn07 and the SAC305 solder joints. The rougher the surface of the solder–substrate interface, the more difficult it is for the crack to propagate. This major difference in the morphology of the intermetallic layer in SAC0307-Mn07 solders could also improve their resistance against a cyclic external load.

Although the SAC305 outperformed the SAC0307-Mn07 solder alloy in strength in an as-reflowed state, this does not necessarily mean that the long-term reliability of the SAC0307-Mn07 solder alloy is also lower. The softer SAC0307-Mn07 solder alloy could be more resistive against vibration and cyclic thermal load. Therefore, the reliability investigation by accelerated lifetime tests of the SAC0307-Mn07 solder joints will be the focus of future research.

## Figures and Tables

**Table 1:** Cooling rates over the different pressure values of the air stream regulator.

**Pressure, ×10^5^ Pa**	0.4	0.8	1.2	1.6	2	2.4	3.2	4.4	7
**Cooling rate** **from 240 down to 230 °C, K/s**	2.7	3.3	3.9	4.8	5.4	6.3	7.6	8.5	14.7

The cooling data were acquired by averaging ten measurements at each cooling rate, and the relative repeatability was ~1%.
